# Magnitude, associated factors, and immediate outcomes of nonreassuring fetal heart rate status among laboring mothers in Ethiopia: a systematic review and meta-analysis

**DOI:** 10.1016/j.xagr.2026.100620

**Published:** 2026-02-26

**Authors:** Gizachew Yilak, Bogale Molla, Befkad Derese Tilahun, Biruk Beletew Abate, Tegene Atamenta Kitaw, Aychew Kassie, Addisu Getie, Besfat Berihun Erega, Mulat Ayele, Eyob Shitie Lake

**Affiliations:** Department of Nursing, College of Health Sciences, Woldia University, Woldia, Ethiopia (Yilak, Molla, Tilahun, Abate, Kitaw, Kassie); Department of Nursing, College of Health Sciences, Debre Markos University, Debre Markos, Ethiopia (Getie); Department of Midwifery, College of Health Sciences, Debre Tabor University, Debre Tabor, Ethiopia (Erega); Department of Midwifery, College of Health Sciences, Woldia University, Woldia, Ethiopia (Ayele, Lake)

**Keywords:** Non-Reassuring Fetal Heart Rate, labouring mothers, Ethiopia, systematic review, meta-analysis

## Abstract

**Objectives:**

To assess the magnitude, associated factors, and immediate outcomes of nonreassuring fetal heart rate status (NRFHRS) among laboring mothers in Ethiopia.

**Design:**

Systematic review and meta-analysis.

**Data Sources:**

Data were extracted from published articles identified by searching using major bibliographic databases such as PubMed/Medline, Cochrane Library, Virtual Health Library (VHL) Regional Portal, HINARI (research4life), and Google Scholar.

**Eligibility Criteria:**

The meta-analysis included all observational studies on NRFHRS among laboring mothers in Ethiopia published in English between 2010 and 2024. Unpublished studies were also considered, and studies without abstracts or full texts were excluded.

**Data Extraction and Synthesis Methods:**

Using PRISMA standards, we systematically reviewed and meta-analyzed articles from PubMed, Cochrane Library, and Google Scholar. Q and I^2^ tests were used to assess heterogeneity across studies. To evaluate the national magnitude and effect size of the linked covariates, a weighted inverse variance random effects model was used. Funnel plots and Egger regression tests were used to examine publication bias. Sensitivity analysis was also performed to determine the impact of the studies.

**Results:**

The analysis included 10 studies with 5949 participants used in the analysis. The pooled magnitude of nonreassuring fetal heart rate (NRFHR) in Ethiopia was 23.29% (14.89, 31.69) augmentation of labor (AOR=3.8; 95% CI: 2.51–5.10; I^2^=0.00%; *P*=.98), being primiparas (AOR=2.33; 95% CI: 1.84–2.84; I^2^=92.66%; *P*=.00), meconium stained amniotic fluid (AOR=5.97; 95% CI: 3.01–8.93; I^2^=83.31%; *P*=.00), mothers who are not referred (AOR=2.65; 95% CI: 3.01–8.93; I^2^=98.21%; *P*=.00), and no antenatal follow up (AOR=4.08; 95% CI: 2.79–5.37; I^2^=97.46%; *P*=.00) respectively were significantly associated with nonreassuring fetal heart rate in Ethiopia.

**Conclusion:**

The magnitude of the NRFHR in Ethiopia remains high, worsened by factors such as labor augmentation, primiparity, meconium-stained amniotic fluid, and lack of antenatal care follow-up, which are associated with adverse outcomes. These include heightened risks of neonatal intensive care unit admission, low Appearance, Pulse, Grimace, Activity, and Respiration scores, perinatal death, and a greater likelihood of needing caesarean section. The critical need for vigilant monitoring and timely intervention is evident to enhance the health of maternal and neonatal outcomes in such cases.

**AJOG Global Reports at a Glance**

**What is already known?**

• Nonreassuring fetal heart rate status (NRFHRS) in Ethiopia has a significant impact on both neonatal and maternal health.
• The magnitude of the NRFHR in Ethiopia is substantial and is influenced by multiple contributing factors.

**What are the new findings?**

• To date, no study has focused on assessing the pooled magnitude, associated factors, and immediate outcomes of NRFHRS among laboring mothers in Ethiopia. Accordingly, the current study found that about one-fourth of laboring mothers in Ethiopia experienced nonreassuring fetal heart rate.
• Major outcomes of NRFHRS include heightened risk of neonatal intensive care unit admission, low APGAR scores, perinatal death, and an increased likelihood of requiring a caesarean section.
• Factors such as labor augmentation, primiparity, presence of meconium-stained amniotic fluid, and inadequate antenatal care follow-up were significantly associated with nonreassuring fetal heart rate.

**What do the new findings imply?**

• The current study provides a clear magnitude of NRFHRS among laboring mothers in Ethiopia. These data can serve as a reference point for future research endeavors and for the formulation of targeted strategies, policies, and programs. The need for interventions to improve maternal and neonatal health outcomes in scenarios involving NRFHR is apparent.
• The present study revealed a notably high magnitude of NRFHRS compared to previous research, potentially influenced by study design variations, including a single study conducted in China. Discrepancies in cultural practices, study locations, timeframes, sociodemographic characteristics, and variations in healthcare service quality could contribute to these differing findings.

## Introduction

Fetal heart rate (FHR) is categorized into Category I (110–160 beats per minute (bpm), variability of 5 to 25 bpm, and no decelerations), Category II (do not fall into either Category I or III), and Category III (no baseline FHR variability followed by bradycardia, recurrent late decelerations, or recurrent variable decelerations).^1^ Nonreassuring fetal heart rate status (NRFHRS) is defined as abnormal fetal heart rate monitoring, including irregular fetal heart rate (FHR) tone (fetal tachycardia or bradycardia), abnormal variability (no, minimal, and/or marked variability), and repeated fetal heart rate deceleration (late deceleration, variable deceleration, and prolonged deceleration).^2^^,^^3^

Based on the literature, the magnitude of the NRFHRS can vary significantly across different countries and healthcare settings: 21.2% in Israel, 11.5% in China, and 30.7% in Thailand, reflecting differences in obstetric practices, patient populations, and healthcare systems.^4^, ^5^, ^6^ In Africa, the prevalence of NRFHRS is 8.9% in Nigeria, 21.16% in South Gonder, Ethiopia, 18.6% in Addis Ababa, Ethiopia, 12.2% in Felege Hiwot Comprehensive Specialized Hospital, and 15.1% in Finote Selam General Hospital, Ethiopia.^7^, ^8^, ^9^, ^10^, ^11^

NRFHRS is responsible for 2.1 million early newborn fatalities and 1.2 million stillbirths worldwide; 98% of these deaths and stillbirths occur in low- and middle-income nations, and 77% occur in sub-Saharan Africa and South Asia.^12^, ^13^, ^14^ NRFHRS can also result in a number of major health problems, including hearing and vision impairment, seizures, necrotizing enterocolitis (NEC), epilepsy, mental retardation, regular prolonged weeping, partial or complete brain damage, cerebral palsy paralysis, nerve damage, and hypoxia.^15^, ^16^, ^17^, ^18^ In addition, it is a significant factor in the decision to use instrumental and surgical interventions during childbirth, accounting for 36% of vacuum delivery and 47.1% to 58% of all cesarean deliveries,^19^^,^^20^ and it is associated with resuscitation at birth, neonatal intensive care unit (NICU) admission, and length of hospitalization worldwide.^21^

Prolonged rupture of the membrane, low birth weight, induction or augmentation of labor, abnormal fetal presentation, use of anesthesia, antepartum hemorrhage, intrauterine growth restriction, amniotic fluid disorders, intrauterine growth restriction, maternal medical illnesses, and being a prime gravida are some of the risk factors associated with NRFHRP, according to various studies conducted worldwide.^4^^,^^8^^,^^11^^,^^22^

In Africa, several strategies have been utilized to decrease the magnitude and adverse effects of the NRFHRS. These include catchment-based reproductive, maternal, newborn, and child health (RMNCH) mentoring; basic emergency obstetric and newborn care; comprehensive emergency obstetric and newborn care; and continuous electronic fetal monitoring.^23^ Despite those efforts, 81% of neonatal mortality occurs in Africa and Asia,^24^ and NRFHR accounts for 3.7% of caesarean delivery in the continent,^22^ 7.5% of caesarean delivery rate in Ethiopia.^9^ However, the reported findings are inconsistent, and to the best of our knowledge, no systematic review or meta-analysis has been conducted to address these conflicting results in Ethiopia. Therefore, this systematic review and meta-analysis aimed to examine the magnitude, associated factors, and immediate outcomes of NRFHRS among laboring mothers in Ethiopia.

## Methods

### Reporting

The findings of this review are presented in accordance with the Preferred Reporting Items for Systematic Review and Meta-Analysis (PRISMA) statement recommendations (PRISMA Checklist and Supplemental Table 1).

### Searching strategy and information sources

We found papers from PubMed, the Cochrane Library, and Google Scholar that provided data on the magnitude, potential risk factors, and immediate birth outcome for NRFHBR in Ethiopia. To retrieve additional publications, the search used Medical Subject Headings (MeSH) terms and keywords, combinations, and snowball searching in the reference list of papers found through the database search. Articles with missing or incorrect data were resolved by contacting the corresponding author. Unpublished studies were obtained from the official websites of international and local organizations and universities.

Medical Subject Headings (MeSH) terms and keywords were used to conduct the search. “(Prevalence OR (Prevalence[MeSH Terms]) magnitude OR magnitude [MeSH Terms]) or epidemiology) OR (epidemiology [MeSH Terms]) AND (causes OR (causes [MeSH Terms]) determinants OR (determinants [MeSH Terms]) (related factors) OR (related factors [MeSH Terms]) OR predictors OR (predictors[MeSH Terms]) OR (risk factors) OR (risk factors [MeSH Terms]) OR (none reassuring fetal heart rate patter (NRFHRP) OR (fetal heart rate patter [MeSH Terms]) (Fetal birth outcome OR (none reassuring fetal heart rate patter birth outcome [MeSH Terms]) OR (fetal birth oucome OR (neonatal birth outcome [MeSH Terms]) OR (fetal heartbeat monitoring) OR (abnormal fetal heartbeat patter [MeSH Terms]) AND (Ethiopia)” (additional file 2) Moreover, the searching strategy that had been used for Google Scholar was illustrated (additional file 3). The search date was June 2024.

We also searched the reference lists of the remaining studies to identify new studies relevant to this review. The criteria for study selection and eligibility to remove duplicate studies were exported to the reference manager program EndNote, version 21. Before retrieving the full-text publications, two investigators (GY and ESL) independently assessed the selected studies using their titles and abstracts. We further screened the full-text papers using prespecified inclusion criteria. Disagreements regarding the final selection of studies to be included in the systematic review and meta-analysis were discussed by additional reviewers (BM, BDT, BBA, TAK, BBE, AG, AK, and MA) during a consensus meeting.

### Inclusion and exclusion criteria

All observational studies were included in the meta-analysis. Research published in English between 2005 and 2024 in Ethiopia has examined the Magnitude, Associated Factors, and Immediate Outcomes of NRFHRS among laboring mothers in Ethiopia: a systematic review and meta-analysis. Unpublished studies on nonreassuring fetal heart rates (NRFHRs) have also received attention. The analysis did not include editorials, anonymous reports, qualitative studies, or citations without an abstract or a full text. Studies that failed to disclose noteworthy findings were excluded. NRFHRS among laboring mothers in Ethiopia during the data collection period met the inclusion and exclusion criteria of the included studies.

### Quality assessment

After integrating the database search results, duplicate articles were deleted using EndNote (version 21). A quality appraisal checklist developed by the Joanna Briggs Institute (JBI) was employed.^25^^,^^26^ The quality of the studies was evaluated by four independent writers. Appraisal was repeated using trading notes. As a result, one study was evaluated by two authors. Any disagreement between the reviewers was resolved by averaging the scores. Studies were considered low-risk or good quality if they scored 5 or higher for all studies and were included.^25^^,^^26^ However, studies with a score of 4 or lower were considered high risk or of poor quality and were not included.

### Data extraction

The data extraction form that the authors created was an Excel file containing the following information: name of the author, year of publication, research region, study design, sample size, immediate outcomes of NRFHR, magnitude of NRFHRP, and categories of factors that were reported. Four papers were randomly selected to test the data-extraction sheet. The extraction method was modified after the experiment using a template. Two authors used an extraction form to extract the data. The accuracy of the data was verified separately by the third, fourth, and fifth authors. If necessary, discussions with a third and fourth reviewer helped settle any disputes that arose among the reviewers. Cross-referencing the data with the included papers allowed the correction of data errors. If incomplete data were obtained, the study was excluded after two email attempts to contact the corresponding author.

### Outcome measurement

NRFHRS: Considered when one or more of the following (Tachycardia or bradycardia for more than 10 minutes), reduced FHR variability, decelerations, and absence of accelerations) occur in the intrapartum period.^27^^,^^28^

A baseline fetal heart rate status: normal between 110 and 160 bpm, Tachycardia >160 bpm, and bradycardia.^27^^,^^28^

Immediate birth outcomes of NRFHRS: outcomes include (Appearance, Pulse, Grimace, Activity, and Respiration (APGAR) score at first minute and fifth minutes, still birth, need for ventilation [asphyxia], and need for neonatal NICU admission), within 5 minutes after delivery.^28^

### Statistical analysis

After extracting the data in Microsoft Excel format, we loaded it into STATA version 17.0, a statistical software for further analysis. Standard error was computed for each study using a binomial distribution formula. A random-effects meta-analysis was used to pool the overall magnitude of the NRFHRS.^29^ Forest plots were used to display the pooled magnitude of NRFHRS with 95% confidence intervals (CI) and odds ratios (OR) with 95% CI to illustrate the factors related to NRFHRS. Using *P*-values, inverse variance (I^2^), and Cochran’s Q statistics (chi-square) were used to examine the heterogeneity among the studies.^30^

In this study, an I^2^ value of zero indicated true homogeneity, whereas values of 25%, 50%, and 75% represented low, moderate, and high heterogeneity, respectively.^31^^,^^32^ We conducted a random effects model analysis of the data identified as heterogeneous. In addition, subgroup analysis was performed according to individual factors, and immediate birth outcomes from the NRFHRS. When statistical pooling is not possible, nonpooled data are presented in a table form. Sensitivity analysis was employed to determine the effect of a single study on the overall estimation. Publication bias was assessed using funnel plots and, more objectively, using Egger’s regression test.^33^

### Patient and public involvement

Patients and/or the public were not involved in the design, conduct, reporting, or dissemination plans of this research.

## Results

**Figure 1:**
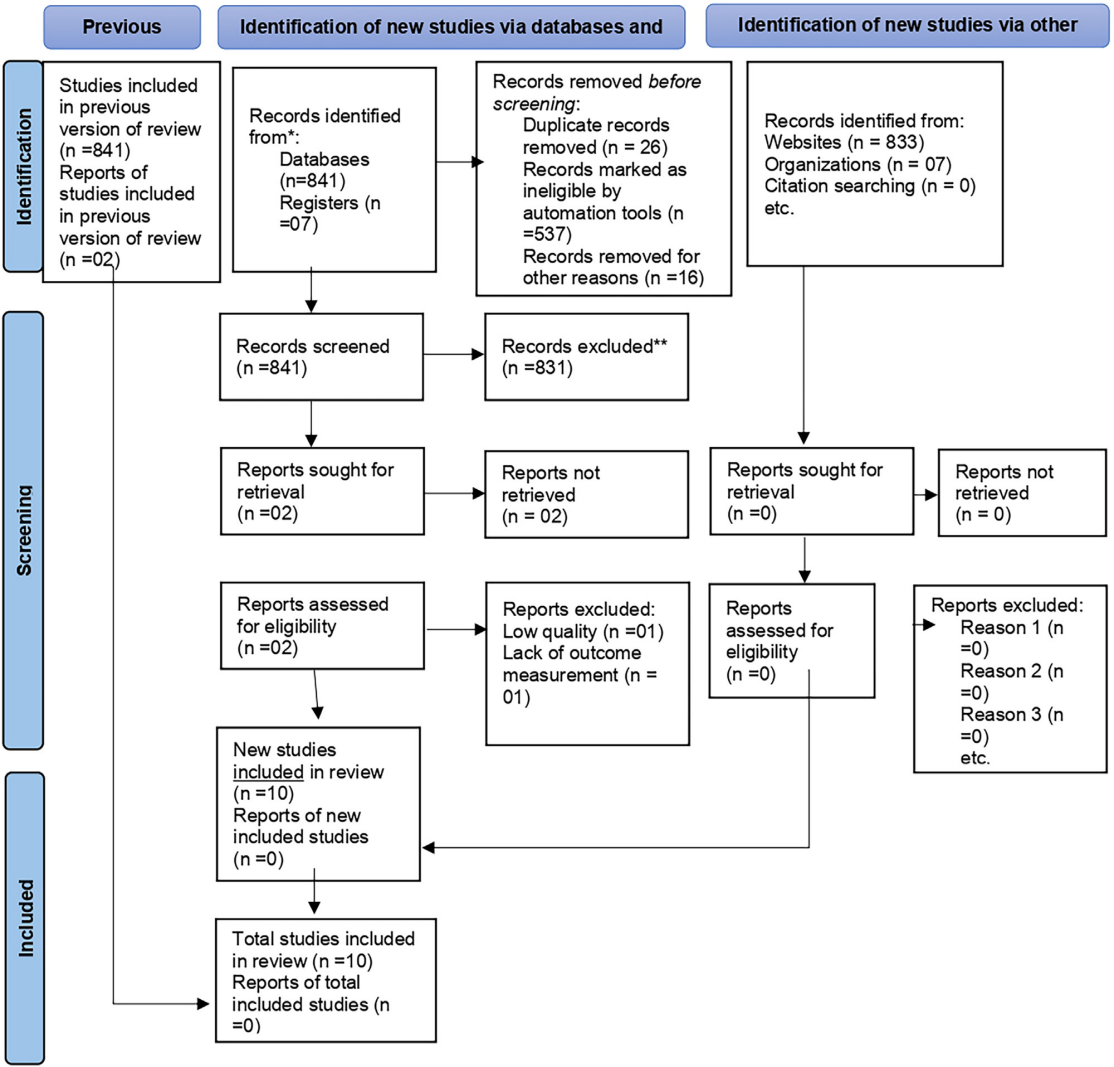
PRISMA flow chart for the selection of systematic reviews and meta-analyses of magnitude, associated factors, and immediate outcomes of nonreassuring fetal heart rate status among laboring mothers in Ethiopia

Study selection: A total of 841 studies were identified using electronic searches (database searches after removing duplicates), and 563 studies were retrieved between 2005 and 2024, of which 537 were rejected by reading only the titles. Of the remaining 26 studies, 12 were excluded after reading abstracts. Finally, 14 studies were screened for full-text review, and 10 articles (n=5949 study participants) were selected for the magnitude, associated factors, and immediate outcomes of NRFHRS among laboring mothers analysis (Figure 1).

Yilak. Magnitude, associated factors, and immediate outcomes of nonreassuring fetal heart rate status among laboring mothers in Ethiopia. AJOG Glob Rep 2025.

### Characteristics of included studies

Table summarizes the characteristics of the 10 included studies in the systematic review and meta-analysis.^8^^,^^9^^,^^11^^,^^23^^,^^34^, ^35^, ^36^, ^37^, ^38^, ^39^ Four studies were found in the Amhara region,^8^^,^^11^^,^^23^^,^^35^ one in southern nationalities,^34^ four in the Oromia region,^36^, ^37^, ^38^, ^39^ and one in Addis Ababa city.^34^ Most of the studies were published between 2014 and 2021. This study included 257^39^ to 1379^9^ participants (Supplemental Table 2).

**Table:** Factors associated with NRFHRS among laboring mothers in Ethiopia

Variables	Odds ratio (95%CI)	Author (reference)	Year	Pooled AOR (95%CI)	I^2^ (*P*-value)
Augmentation of labor	3.664 (0.37–6.96)	Kassahun^11^	2020	3.8 (2.5–5.10)	000%
	3.78 (1.4–6.15)	Belete^8^	2022		
	3.51 (1.05–5.97)	Minalbat^35^	2022		
	4.2 (1.74–6.65)	Misba^38^	2023		
Being primipara	2.722 (2.42–3.02)	Kassahun^11^	2020	2.33 (1.84–2.82)	92.66%
	1.86 (1.64–2.08)	Belete^8^	2022		
	1.95 (1.70–2.20)	Minalbat^35^	2022		
	2.82 (2.51–3.13)	Ruth^36^	2023		
Meconium- stained amniotic fluid	1.89 (0.64–3.14)	Asnake^34^	2023	5.97 (3.01–8.93)	83.1%
	6.491 (2.83–10.16)	Kassahun^11^	2020		
	14.13 (8.94–19.32)	Belete^8^	2022		
	6.412 (2.77–10.05)	Minalbat^35^	2022		
	5.32 (2.04–8.60)	Ruth^36^	2023		
	4.5 (1.55–7.45)	Misba^38^	2023		
Mothers who are not referred	1.58 (1.08–2.08)	Asnake^34^	2023	2.65 (1.51–3.79)	98.21%
	2.83 (2.54–3.12)	Kassahun^11^	2020		
	1.95 (1.69–3.32)	Belete^8^	2022		
	4.2 (3.94–4.46)	Misba^38^	2023		
No antenatal follow-up	3.46 (3.31–3.61)	Asnake^34^	2023	4.08 (2.79–5.37)	92.91%
	4.78 (4.11–5.45)	Ruth^36^	2023		

**Figure 2:**
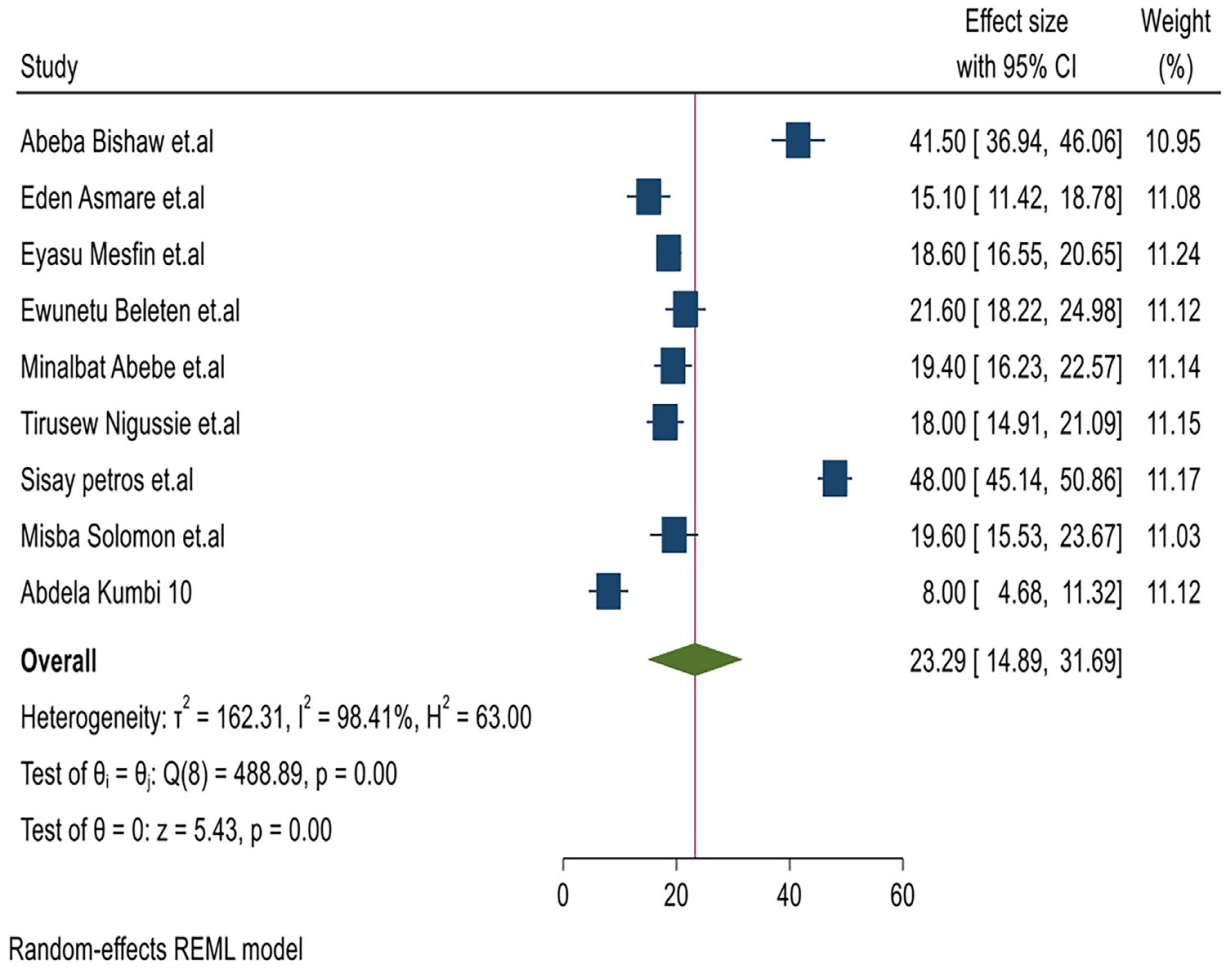
Frost plot on the pooled magnitude, associated factors, and immediate outcomes of nonreassuring fetal heart rate status among laboring mothers in Ethiopia

Meta-analysis of magnitude, associated factors, and immediate outcomes of NRFHRS among laboring mothers in Ethiopia. Most of the studies (n=9) reported the magnitude of NRFHRS among laboring mothers in Ethiopia.^8^^,^^9^^,^^11^^,^^23^^,^^34^^,^^35^^,^^37^, ^38^, ^39^ The magnitude of NRFHRS among laboring mothers in Ethiopia ranged from 8.0%^39^ up to 48%.^37^ The random-effects model analysis from those studies revealed that the pooled magnitude of NRFHRS among laboring mothers in Ethiopia was 23.29% (95% CI, 14.89–31.69; I^2^=98.41%; *P*<.001) (Figure 2). The I-squared (I^2^) and *P*-values also confirmed the presence of heterogeneity. Regarding publication bias for the magnitude of NRFHRS among laboring mothers in Ethiopia, funnel plot analysis showed an asymmetrical distribution. We employed a leave-one-out sensitivity analysis to identify the potential source of heterogeneity in the pooled estimate of NRFHRS among laboring mothers in Ethiopia.

Yilak. Magnitude, associated factors, and immediate outcomes of nonreassuring fetal heart rate status among laboring mothers in Ethiopia. AJOG Glob Rep 2025.

### Sensitivity analysis

In this systematic review and meta-analysis, a leave-one-point sensitivity analysis conducted using the random-effects model suggested that none of the points were estimates outside the overall 95% confidence interval, confirming that there was no influential study (Supplemental Table 3).

### Factors associated with magnitude of NRFHRS among laboring mothers in Ethiopia

#### Augmentation of labor

**Figure 3:**
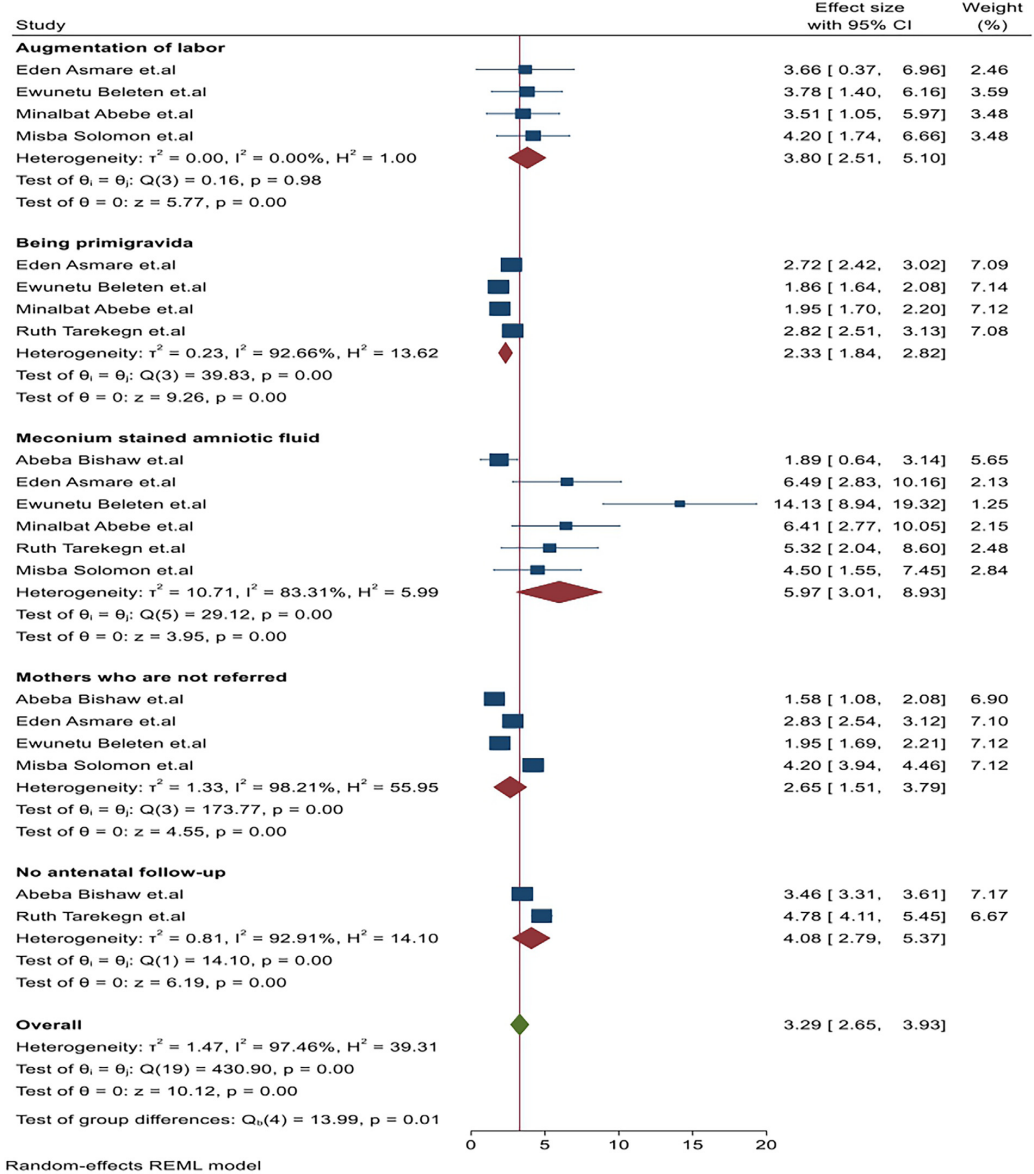
Frost plot of the pooled associated factors of nonreassuring fetal heart rate status among laboring mothers in Ethiopia

Among the included studies, four^8^^,^^11^^,^^35^^,^^38^ identified factors associated with NRFHRS among laboring mothers in Ethiopia (Table). These studies revealed a significant association between labor augmentation and NRFHRS among laboring mothers in Ethiopia. Notably, the study with the highest risk factor reported an (AOR) of 4.2 (1.74, 6.66),^38^ while the lowest risk factor had an AOR of 3.5 (1.05, 5.97)^35^ compared to laboring mothers without augmentation (Table). In terms of heterogeneity testing for labor augmentation, the Galbraith plot displayed heterogeneity. When combining the results of the four studies, the forest plot indicated an overall estimated AOR for augmentation of labor of 3.8 (95% CI: 2.51–5.10); I^2^=00%; *P*=.98. This study was supported by Beer Sheva, Israel.^40^ The I-squared (I^2^) and *P*-values also confirmed the absence of heterogeneity (Figure 3). Regarding publication bias for augmentation of labor, funnel plot analysis showed an asymmetrical distribution. We employed a leave-one-out sensitivity analysis to identify the potential source of heterogeneity in the pooled estimate of mothers with augmentation of labor as a risk factor for NRFHRS among laboring mothers in Ethiopia (Supplemental Figure 1).

Yilak. Magnitude, associated factors, and immediate outcomes of nonreassuring fetal heart rate status among laboring mothers in Ethiopia. AJOG Glob Rep 2025.

#### Being primipara mothers

Four studies have identified a significant association between primipara status and NRFHRS among laboring mothers in Ethiopia. Among these studies, the highest risk factor reported an adjusted odds ratio (AOR) of 2.72 (2.42, 3.02),^11^ while the lowest risk factor had an AOR of 1.86 (1.64, 2.08)^8^ compared to multipara mothers (Table).

In terms of the heterogeneity test for being primipara, the Galbraith plot displayed heterogeneity when combining the results of the four studies. The forest plot showed that the overall estimated AOR for being primipara was 2.33 (95% CI: 1.84–2.82; I^2^=92.66%; *P*=.001). The I^2^ statistics and *P*-values also confirmed the presence of heterogeneity. Regarding publication bias for primiparas, the funnel plot analysis revealed an asymmetrical distribution. To investigate potential sources of heterogeneity in the analysis of the pooled estimate of primipara mothers as a risk factor for NRFHRS among laboring mothers in Ethiopia, a leave-one-out sensitivity analysis was conducted. The results of this sensitivity analysis indicated that our findings were not driven by any single study.

#### Meconium-stained amniotic fluid

Six studies have identified a significant association between meconium-stained amniotic fluid and NRFHRS among laboring mothers in Ethiopia. Among these studies, the highest risk factor reported an AOR of 14.13 (8.94, 19.32),^8^ while the lowest risk factor had an AOR of 1.89 (0.64, 3.14).^34^ Combining the results of the six studies, the forest plot indicated that the overall estimated AOR for meconium-stained amniotic fluid was 5.97 (95% CI: 3.01–8.91; I^2^=83.31%, *P*=.00). The I^2^ statistic and *P*-value also suggest homogeneity (Table). The I^2^ statistic and *P*-values also confirmed the presence of heterogeneity (Figure 3). Regarding publication bias for primiparas, the funnel plot analysis revealed an asymmetrical distribution. To investigate potential sources of heterogeneity in the analysis of the pooled estimate of primipara mothers as a risk factor for NRFHRS among laboring mothers in Ethiopia, a leave-one-out sensitivity analysis was conducted. The results of this sensitivity analysis indicated that our findings were not driven by any single study.

#### Mothers who are not referred

Four studies have identified a significant association between mothers who are not referred and NRFHRS among laboring mothers in Ethiopia. Among these studies, the highest risk factor reported an AOR of 4.2 (3.94, 4.46),^38^ while the lowest risk factor had an AOR of 1.58 (1.08, 2.08).^34^ Compared to those who were referred (Figure 3). Pooling the results of the four studies, the forest plot indicated that the overall estimated AOR for mothers who were not referred was 2.65 (95% CI: 1.51–3.79; I^2^=98.21%; *P*=.00). I^2^ statistics and *P*-values also indicated homogeneity (Figure 3). In terms of the heterogeneity test, the Galbraith plot displayed heterogeneity among studies. Regarding publication bias, the funnel plot showed asymmetrical distribution. The Egger’s regression test resulted in a *P*-value of .00, suggesting the presence of publication bias. To identify potential sources of heterogeneity in the pooled estimate of mothers who are not referred to as a risk factor for NRFHRS among laboring mothers in Ethiopia, a leave-one-out sensitivity analysis was conducted. The results of this sensitivity analysis demonstrated that our findings did not rely on a single study (Supplemental Figure 1).

#### No antenatal follow-up

Two studies have identified a significant association between no antenatal follow-up and NRFHRS among laboring mothers in Ethiopia. The highest risk factor reported an AOR of 4.78 (4.11–5.45),^36^ while the lowest risk factor had an AOR of 3.46 (3.31, 3.61).^34^ Compared to those who underwent antenatal follow-up (Figure 3). Regarding the heterogeneity test, the Galbraith plot showed heterogeneity, and combining the results of the two studies, the forest plot showed that the overall estimate of the AOR for no antenatal follow-up was 4.08 (95% CI: 2.79, 5.37; I^2^=92.9%; *P*=.00). The I^2^ statistics and *P*-values also confirmed the presence of heterogeneity (Figure 3). Regarding publication bias for nonantenatal follow-up, funnel plot analysis revealed an asymmetrical distribution. To investigate potential sources of heterogeneity in the analysis of the pooled estimate of no antenatal follow-up mothers as a risk factor for NRFHRS among laboring mothers in Ethiopia, a leave-one-out sensitivity analysis was conducted. The results of this sensitivity analysis indicated that our findings were not driven by any single study (Supplemental Figure 1).

### NRFHR and birth outcome among laboring mothers in Ethiopia

#### Admission to NICU

**Figure 4:**
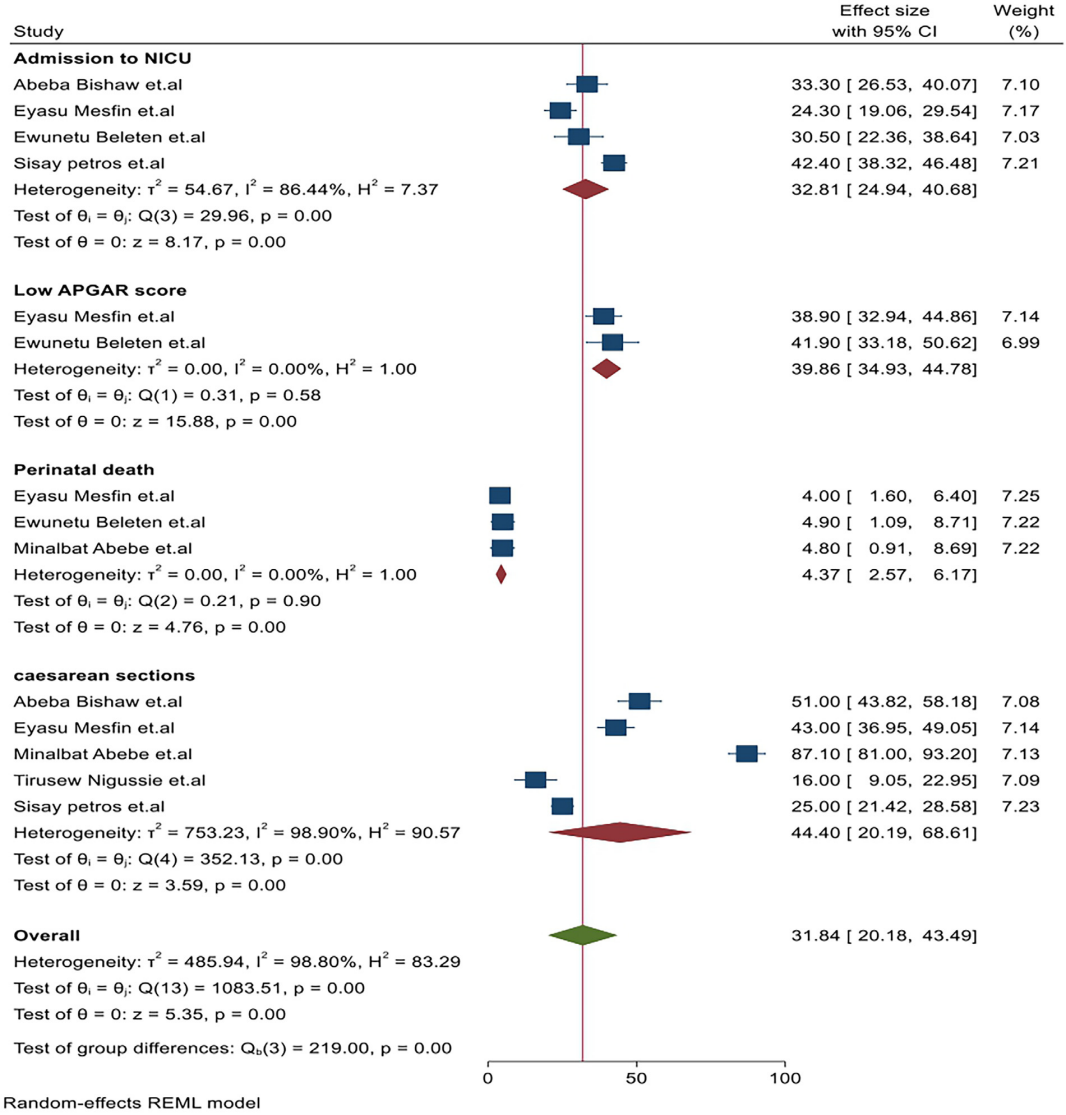
Frost plot of the pooled immediate outcomes of nonreassuring fetal heart rate status among laboring mothers in Ethiopia

Four studies have highlighted a significant number of babies born with NRFHR among laboring mothers in Ethiopia who were admitted to the NICU. The highest burden was reported as 42.4 (38.32, 48.46),^37^ while the lowest burden was 24.30 (19.06, 29.54).^9^ Compared to those who have reassuring fetal heart rate among laboring mothers in Ethiopia (Figure 4). Upon conducting the heterogeneity test and combining the results of the four studies, the forest plot illustrated that the overall magnitude of admission to the NICU among NRFHR laboring mothers in Ethiopia was 32.81 (95% CI: 24.94, 40.68; I^2^=86.44%; *P*=.58). The I^2^ statistics validated the presence of heterogeneity (Figure 4). Concerning publication bias related to NICU admission, analysis of the funnel plot revealed an asymmetrical distribution. To explore potential sources of heterogeneity in the analysis of the pooled estimate of Admission to NICU as a potential outcome of NRFHR among laboring mothers in Ethiopia, a leave-one-out sensitivity analysis was conducted. The results of this sensitivity analysis indicate that our conclusions were not reliant on any single study.

Yilak. Magnitude, associated factors, and immediate outcomes of nonreassuring fetal heart rate status among laboring mothers in Ethiopia. AJOG Glob Rep 2025.

#### Perinatal death

Three studies have highlighted a significant association between babies born with NRFHR among laboring mothers in Ethiopia and an increased likelihood of perinatal death. The highest burden was reported as 4.90 (1.09, 8.71),^8^ while the lowest burden was 4.00 (1.60, 6.40).^9^ Compared to those who did not have an NRFHR among laboring mothers in Ethiopia (Figure 4). Regarding the heterogeneity test and upon combining the results of the three studies, the forest plot showed that the overall incidence of perinatal death among NRFHR cases among laboring mothers in Ethiopia was 4.37 (95% CI: 2.57, 6.17; I^2^=0.00%; *P*=.90). The I^2^ and *P*-values also confirmed the absence of heterogeneity (Figure 3). Regarding publication bias for the augmentation of labor, funnel plot analysis showed an asymmetrical distribution. We employed a leave-one-out sensitivity analysis to identify the potential source of heterogeneity in the pooled estimate of mothers with Augmentation of labor as a risk factor for NRFHRS among laboring mothers in Ethiopia.

#### Caesarean section

Five studies have identified a significant association between babies born with NRFHR among laboring mothers in Ethiopia and an increased likelihood of caesarean section. The highest burden was reported as 87.10 (81.00, 93.20),^35^ while the lowest burden was 16.00 (9.05, 22.95)^23^ when we compared to those who did not experience NRFHR among laboring mothers in Ethiopia (Figure 4). Regarding the heterogeneity test and upon combining the results of the studies, the forest plot illustrated that the overall rate of caesarean section among NRFHR cases among laboring mothers in Ethiopia was 44.40 (95% CI: 20.19, 68.61; I^2^=98.9%; *P*=.00). The I^2^ and *P*-value statistics confirmed the presence of heterogeneity (Figure 4). Concerning publication bias related to caesarean sections, the analysis of the funnel plot revealed an asymmetrical distribution. To explore potential sources of heterogeneity in the analysis of the pooled estimate of caesarean section as a potential outcome of NRFHR among laboring mothers in Ethiopia, a leave-one-out sensitivity analysis was conducted. The results from this sensitivity analysis indicated that our conclusions were not reliant on any single study.

## Discussion

Although the associations identified in this meta-analysis do not establish causality, the pooled national estimates provide a clearer epidemiologic understanding of NRFHRS in Ethiopia, where previous findings were inconsistent and regionally fragmented. This systematic review and meta-analysis delved into the prevalence, associated factors, and immediate outcomes of NRFHR among laboring mothers in Ethiopia, incorporating 10 studies in its final analysis, with 9 studies reporting NRFHR prevalence. The pooled prevalence of NRFHR among laboring mothers in Ethiopia was 23.29% (95% CI: 14.89%–31.69%), consistent with studies from Tel Aviv, Israel (21.2%),^4^ a low-resource Nigerian setting (24.05%),^7^ and Bangladesh (16.4%).^18^ These collective findings emphasize the significant concern of NRFHR among laboring mothers in Ethiopia, showing a prevalence rate similar to that observed in other nations. However, it was higher than that reported in a study conducted in China (11.5%).^5^ Possible reasons for these differences could include variations in the nature of the study, such as the study conducted in China in a single study, as well as differences in cultural practices and disparities in the study area, period, and sociodemographic and healthcare service variations between Ethiopia and China, which might have also contributed to the observed variation.^5^

A systematic review and meta-analysis revealed that factors such as augmentation of labor, being primipara, meconium-stained amniotic fluid, delayed referral to health facilities, and lack of antenatal follow-up were significant risk factors contributing to an elevated magnitude of NRFHR patterns among laboring mothers in Ethiopia. Additionally, outcomes potentially associated with fetuses exhibiting NRFHR patterns among laboring mothers in Ethiopia included admission to the NICU, low APGAR scores, perinatal mortality, and the likelihood of undergoing a caesarean section.

As per the findings of this systematic review and meta-analysis, there exists a significant association between the augmentation of labor and the presence of NRFHR patterns among laboring mothers in Ethiopia. This study indicates that the odds of experiencing NRFHR patterns were three times higher among women who underwent labor augmentation than among those who did not receive such augmentation during childbirth. This may be due to the potential impact of labor augmentation on uterine contractions and fetal well-being. Augmentation methods, such as the use of oxytocin, can sometimes lead to hyperstimulation of the uterus, which in turn can affect fetal heart rate patterns. Additionally, labor augmentation may increase stress on both the mother and the fetus, potentially influencing the occurrence of NRFHR patterns during labor.^11^

In this systematic review, it was observed that the probability of encountering NRFHR patterns was nearly two times greater among primiparous mothers than among multiparous mothers. This finding was supported by a study conducted in China, Beer Sheva, Israel, and Nigeria.^5^^,^^7^^,^^40^ The higher likelihood of encountering NRFHR patterns among primiparous mothers than among multiparous mothers could be attributed to factors such as differences in childbirth experience, maternal stress levels, physiological responses to labor, and variations in uterine dynamics between first-time mothers and those who had previously given birth. Additionally, variations in obstetric practices, maternal age, and overall health status may also have contributed to this observed difference.^6^

In this systematic review, the odds of NRFHRP were almost six times higher among participants whose mothers had meconium-stained amniotic fluid. This finding was supported by a study conducted in low resource Nigerian setting and Beer Sheva, Israel.^7^^,^^40^^,^^41^ Meconium staining in amniotic fluid can indicate fetal distress, as it may suggest that the fetus has passed stool in utero, which can sometimes be a sign of hypoxia or other issues affecting the fetus. The presence of meconium-stained amniotic fluid can trigger healthcare providers to closely monitor fetal heart rate for signs of distress, leading to a higher likelihood of observing NRFHR patterns in such cases.^42^

The odds of NRFHRP were four times higher among participants who did not have antenatal care follow-up at home than among those who did. This finding was supported by a study conducted in low resources setting in Nigeria.^7^ Regular antenatal care plays a crucial role in monitoring the health and well-being of both the mother and the fetus throughout pregnancy. Without proper antenatal care, potential complications or issues that could affect fetal well-being may go undetected, leading to an increased risk of NRFHR patterns during labor. Additionally, the absence of antenatal care may result in missed opportunities for interventions or treatments that could help prevent or manage conditions that contribute to NRFHR patterns.^7^

Furthermore, evidence indicates that admission to the NICU may be higher among laboring mothers in Ethiopia with NRFHR patterns compared to those with reassuring fetal heart rate patterns, which is in line with a study conducted in Bangladesh.^18^ This disparity in NICU admissions suggests a potential association between NRFHR patterns during labor and the need for increased neonatal care and monitoring after birth. Evidence suggests that low APGAR scores may be more prevalent among laboring mothers in Ethiopia with NRFHR patterns than among those with reassuring fetal heart rate patterns, in line with a study conducted in Bangladesh.^18^ This observation indicates a potential correlation between NRFHR patterns during labor and lower APGAR scores at birth, highlighting the importance of monitoring fetal well-being during labor to ensure better neonatal outcomes.

Evidence suggests that perinatal death rates may be higher among laboring mothers in Ethiopia with NRFHR patterns compared to those with reassuring fetal heart rate patterns, which is in line with a study conducted in Bangladesh.^18^^,^^43^ This finding underscores the critical importance of monitoring fetal well-being during labor, as NRFHR patterns may be indicative of potential complications that could result in adverse perinatal outcomes, such as perinatal death. Early detection and appropriate management of NRFHR patterns are essential to reduce the risk of adverse perinatal events.

Evidence indicates that the rate of caesarean sections may be higher among laboring mothers in Ethiopia with NRFHR patterns than among those with reassuring fetal heart rate patterns, which is in line with a study conducted in Nigeria.^7^^,^^44^^,^^45^ This observation suggests that NRFHR patterns during labor may increase the likelihood of caesarean delivery. Close monitoring and timely interventions are crucial in cases where NRFHR patterns are detected to ensure the well-being of both the mother and baby during childbirth. Moreover, these findings underscore the importance of vigilant monitoring and timely interventions to improve maternal and neonatal outcomes during labor in Ethiopia.

### Strength and limitations

This study had several methodological strengths. First, it followed a prespecified and structured search strategy and data extraction process according to established systematic review guidelines. Internationally recognized appraisal tools were used to assess the methodological quality of the included studies. Second, subgroup and sensitivity analyses were performed to explore heterogeneity and assess the robustness of pooled estimates across specific factors and immediate neonatal outcomes.

However, several limitations of this study should be acknowledged. The findings were based exclusively on observational studies; therefore, the identified associations between intrapartum factors and NRFHRS should not be interpreted as causal relationships. Additionally, variations in the diagnostic criteria, monitoring methods, and clinical documentation across the included studies may have influenced the pooled estimates. Although this review aimed to generate a national estimate, some regions of Ethiopia were not represented due to a lack of available data, which may limit generalizability. Finally, although this study provides consolidated epidemiological evidence, it does not directly evaluate the effectiveness of specific clinical interventions or management strategies.

## Conclusion and recommendation

Evidence from Ethiopia indicates that NRFHR patterns during labor are prevalent and linked to unfavorable outcomes. This study provides a consolidated national estimate of the NRFHRS and its associated factors, which may support improved clinical awareness and risk recognition in intrapartum care, particularly in resource-limited settings. Laboring mothers facing factors such as labor augmentation, primiparity, meconium-stained amniotic fluid, lack of antenatal care follow-up, and NRFHR patterns face heightened risks of adverse events such as NICU admission, low APGAR scores, perinatal death, and an increased likelihood of needing a caesarean section. These findings emphasize the critical need for vigilant monitoring and prompt interventions to enhance maternal and neonatal outcomes during labor in Ethiopia.

## CRediT authorship contribution statement

**Gizachew Yilak:** Writing – review & editing, Writing – original draft, Visualization, Validation, Supervision, Software, Project administration, Methodology, Investigation, Funding acquisition, Formal analysis, Data curation, Conceptualization. **Bogale Molla:** Writing – review & editing, Writing – original draft, Visualization, Supervision, Funding acquisition, Formal analysis, Data curation. **Befkad Derese Tilahun:** Writing – review & editing, Writing – original draft, Visualization, Supervision, Software, Project administration, Methodology, Investigation, Formal analysis, Data curation, Conceptualization. **Biruk Beletew Abate:** Writing – review & editing, Writing – original draft, Visualization, Software, Resources, Methodology, Formal analysis, Data curation, Conceptualization. **Tegene Atamenta Kitaw:** Writing – review & editing, Writing – original draft, Visualization, Software, Project administration, Methodology, Investigation, Formal analysis, Data curation, Conceptualization. **Aychew Kassie:** Writing – review & editing, Writing – original draft, Visualization, Supervision, Software, Project administration, Methodology, Funding acquisition, Formal analysis, Data curation. **Addisu Getie:** Writing – review & editing, Writing – original draft, Supervision, Software, Project administration, Methodology, Formal analysis, Data curation, Conceptualization. **Besfat Berihun Erega:** Writing – review & editing, Visualization, Validation, Software, Project administration, Methodology, Formal analysis, Conceptualization. **Mulat Ayele:** Writing – review & editing, Writing – original draft, Visualization, Supervision, Software, Project administration, Methodology, Investigation, Formal analysis, Data curation, Conceptualization. **Eyob Shitie Lake:** Writing – review & editing, Writing – original draft, Supervision, Software, Project administration, Methodology, Investigation, Formal analysis, Data curation.

## Appendix Supplementary materials

Supplemental Fig. 1: Funnel plot showing the symmetric distribution of each primary study on the pooled magnitude of Non-Reassuring Fetal Heart Rate Status among Laboring Mothers in Ethiopia. Image, image 1 dummy alt text

Supplemental Table 1: Different databases were searched to find the magnitude, associated factors, and immediate outcomes of NRFHRS among laboring mothers in Ethiopia Supplemental Table 2: Distribution of studies on the magnitude, associated factors, and immediate outcomes of non-reassuring fetal heart rate status among laboring mothers in Ethiopia: a systematic review and meta-analysis 2010–2023. Supplemental Table 3: Sensitivity analysis of the magnitude of NRFHRS among laboring mothers in Ethiopia Image, application 2

## Footnotes

Supplementary material associated with this article can be found, in the online version, at doi:10.1016/j.xagr.2026.100620.
