# Supplementary material for: Magnitude, associated factors, and immediate outcomes of nonreassuring fetal heart rate status among laboring mothers in Ethiopia: a systematic review and meta-analysis
Source: AJOG Glob Rep. 2026 Feb 26;6(2):100620. doi: 10.1016/j.xagr.2026.100620 (PMC13019767; doi:10.1016/j.xagr.2026.100620)
Supplement: Supplementary file 2 — Supplemental Table 1: Different databases were searched to find the magnitude, associated factors, and immediate outcomes of NRFHRS among laboring mothers in Ethiopia Supplemental Table 2: Distribution of studies on the magnitude, associated factors, and immediate outcomes of non-reassuring fetal heart rate status among laboring mothers in Ethiopia: a systematic review and meta-analysis 2010–2023. Supplemental Table 3: Sensitivity analysis of the magnitude of NRFHRS among laboring mothers in Ethiopia [file mmc2.pdf]

Supplementary table 1: Different databases were searched to find articles magnitude, associated factors, and immediate outcomes of non-reassuring fetal heart rate status among labouring mothers in Ethiopia

Databases	Searching terms	Number of studies
MEDLINE/ PubMed	Medical Subject Headings (MeSH) terms and keywords were used to conduct the search. "(Prevalence OR (Prevalence[MeSH Terms]) magnitude OR magnitude [MeSH Terms]) or epidemiology) OR (epidemiology [MeSH Terms]) AND (causes OR (causes [MeSH Terms]) determinants OR (determinants [MeSH Terms]) (related factors) OR (related factors [MeSH Terms]) OR predictors OR (predictors[MeSH Terms]) OR (risk factors) OR (risk factors [MeSH Terms]) OR (none reassuring fetal heart rate patter (NRFHRP) OR (none reassuring fetal heart rate patter birth outcome [MeSH Terms]) OR (fetal birth oucome OR (fetal heartbeat monitoring) OR (abnormal fetal heartbeat patter [MeSH Terms]) AND (Ethiopia)"	380
Google Scholar	(None reassuring fetal heart rate pattern) OR (Fetal birth outcome) OR AND OR AND (related factors) OR predictors OR (risk factors) OR (Ethiopia)"	423
Other databases	"(Prevalence OR (Prevalence) magnitude OR magnitude or epidemiology) OR (epidemiology AND (causes OR (causes) determinants OR (determinants) (related factors) OR (related factors) OR predictors OR (predictors) OR (risk factors) OR (none reassuring fetal heart rate patter (NRFHRP) OR (fetal heart rate patter) AND Ethiopia. "	38
Total retrieved articles		841
Included studies		10

Supplementary Table 2: Distribution of studies on the Magnitude, Associated Factors, and Immediate Outcomes of Non-Reassuring Fetal Heart Rate Status among Labouring Mothers in Ethiopia: A Systematic Review and Meta-Analysis 2010–2023.

Author/Reference	year	Region	study design	sample size	p	Quality score
Belete.E et.al (8)	2022	SNNPS	cross-sectional	448	41.5	10
Kassa EM et al. (9)	2022	Addis Ababa	cross-sectional	364	15.1	9
Kassahun EA et al. (11)	2020	Amhara	cross-sectional	1379	18.6	10
Kebede TN *et al. (23)	2024	Amhara	cross-sectional	571	21.6	9
Asnake AB et al. (34)	2023	Amhara	cross-sectional	598	19.4	10
Minalbat A, et al. (35)	2022	Amhara	cross-sectional	594	18	10
Ruth Tareke et al. (36)	2023	Oromia	case control	215	-	10
Sisay Petros et al. (37)	2015	Oromia	cohort	1173	48	8
Misbah Solomon et al. (38)	2023	Oromia	cross-sectional	365	19.6	9
Dr. Abdela et al. (39)	2014	Oromia	cross-sectional	257	8	10

Supplementary Table 3: Sensitivity analysis of the magnitude of non-reassuring fetal heart rate status among labouring mothers in Ethiopia

Omitted study Effect size	Effect size [95% conf. interval]			P value
Abeba Bishaw et.al	21.056	12.947	29.164	0.000
Eden Asmare et.al	24.315	15.065	33.565	0.000
Eyasu Mesfin et.al	23.888	14.440	33.336	0.000
Ewunetu Beleten et.al	23.506	13.991	33.022	0.000
Minalbat Abebe et.al	23.782	14.315	33.250	0.000
Tirusew Nigussie et.al	23.958	14.543	33.374	0.000
Sisay petros et.al	20.150	13.643	26.657	0.000
Misba Solomon et.al	23.753	14.288	33.217	0.000
Abdela Kumbi	25.204	16.678	33.731	0.000
theta	23.292	14.891	31.693	0.000
